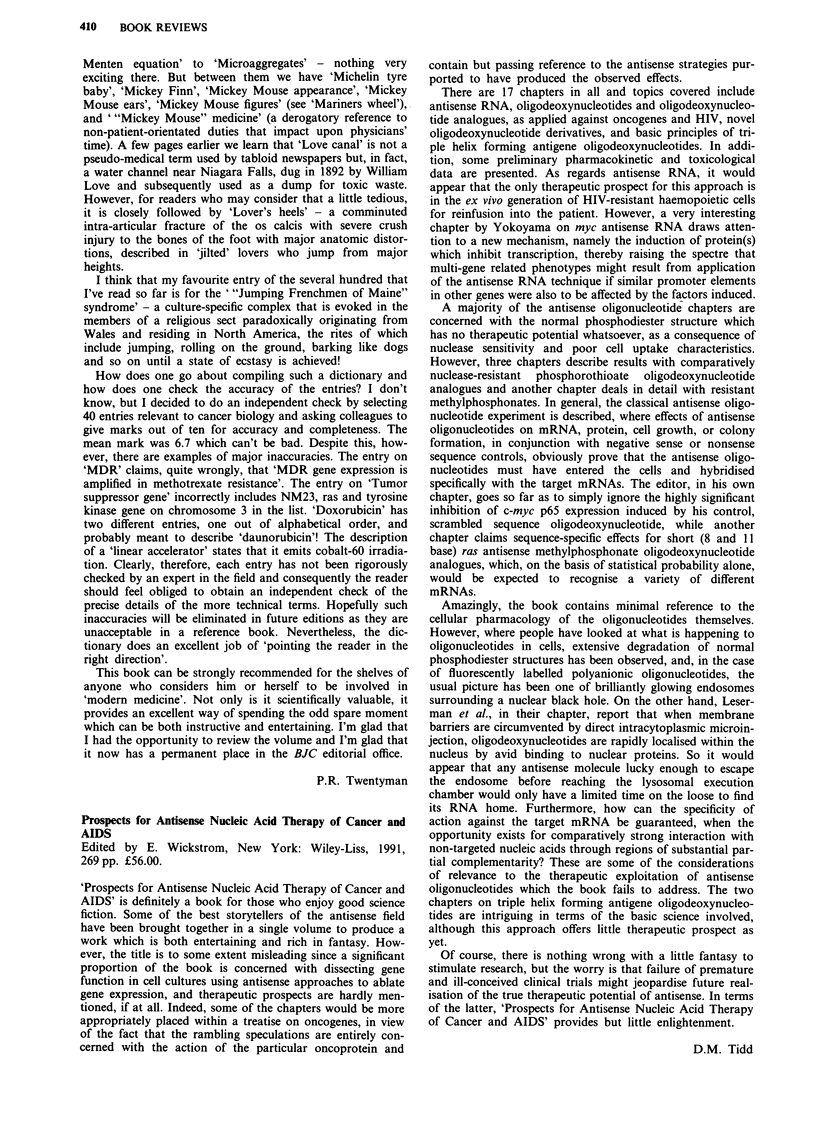# Prospects for Antisense Nucleic Acid Therapy of Cancer and AIDS

**Published:** 1993-02

**Authors:** D.M. Tidd


**Prospects for Antisense Nucleic Acid Therapy of Cancer and AIDS**

Edited by E. Wickstrom, New York: Wiley-Liss, 1991, 269 pp. £56.00.

'Prospects for Antisense Nucleic Acid Therapy of Cancer and AIDS' is definitely a book for those who enjoy good science fiction. Some of the best storytellers of the antisense field have been brought together in a single volume to produce a work which is both entertaining and rich in fantasy. However, the title is to some extent misleading since a significant proportion of the book is concerned with dissecting gene function in cell cultures using antisense approaches to ablate gene expression, and therapeutic prospects are hardly mentioned, if at all. Indeed, some of the chapters would be more appropriately placed within a treatise on oncogenes, in view of the fact that the rambling speculations are entirely concerned with the action of the particular oncoprotein and contain but passing reference to the antisense strategies purported to have produced the observed effects.

There are 17 chapters in all and topics covered include antisense RNA, oligodeoxynucleotides and oligodeoxynucleotide analogues, as applied against oncogenes and HIV, novel oligodeoxynucleotide derivatives, and basic principles of triple helix forming antigene oligodeoxynucleotides. In addition, some preliminary pharmacokinetic and toxicological data are presented. As regards antisense RNA, it would appear that the only therapeutic prospect for this approach is in the *ex vivo* generation of HIV-resistant haemopoietic cells for reinfusion into the patient. However, a very interesting chapter by Yokoyama on *myc* antisense RNA draws attention to a new mechanism, namely the induction of protein(s) which inhibit transcription, thereby raising the spectre that multi-gene related phenotypes might result from application of the antisense RNA technique if similar promoter elements in other genes were also to be affected by the factors induced.

A majority of the antisense oligonucleotide chapters are concerned with the normal phosphodiester structure which has no therapeutic potential whatsoever, as a consequence of nuclease sensitivity and poor cell uptake characteristics. However, three chapters describe results with comparatively nuclease-resistant phosphorothioate oligodeoxynucleotide analogues and another chapter deals in detail with resistant methylphosphonates. In general, the classical antisense oligonucleotide experiment is described, where effects of antisense oligonucleotides on mRNA, protein, cell growth, or colony formation, in conjunction with negative sense or nonsense sequence controls, obviously prove that the antisense oligonucleotides must have entered the cells and hybridised specifically with the target mRNAs. The editor, in his own chapter, goes so far as to simply ignore the highly significant inhibition of c-*myc* p65 expression induced by his control, scrambled sequence oligodeoxynucleotide, while another chapter claims sequence-specific effects for short (8 and 11 base) *ras* antisense methylphosphonate oligodeoxynucleotide analogues, which, on the basis of statistical probability alone, would be expected to recognise a variety of different mRNAs.

Amazingly, the book contains minimal reference to the cellular pharmacology of the oligonucleotides themselves. However, where people have looked at what is happening to oligonucleotides in cells, extensive degradation of normal phosphodiester structures has been observed, and, in the case of fluorescently labelled polyanionic oligonucleotides, the usual picture has been one of brilliantly glowing endosomes surrounding a nuclear black hole. On the other hand, Leserman *et al.*, in their chapter, report that when membrane barriers are circumvented by direct intracytoplasmic microinjection, oligodeoxynucleotides are rapidly localised within the nucleus by avid binding to nuclear proteins. So it would appear that any antisense molecule lucky enough to escape the endosome before reaching the lysosomal execution chamber would only have a limited time on the loose to find its RNA home. Furthermore, how can the specificity of action against the target mRNA be guaranteed, when the opportunity exists for comparatively strong interaction with non-targeted nucleic acids through regions of substantial partial complementarity? These are some of the considerations of relevance to the therapeutic exploitation of antisense oligonucleotides which the book fails to address. The two chapters on triple helix forming antigene oligodeoxynucleotides are intriguing in terms of the basic science involved, although this approach offers little therapeutic prospect as yet.

Of course, there is nothing wrong with a little fantasy to stimulate research, but the worry is that failure of premature and ill-conceived clinical trials might jeopardise future realisation of the true therapeutic potential of antisense. In terms of the latter, 'Prospects for Antisense Nucleic Acid Therapy of Cancer and AIDS' provides but little enlightenment.

D.M. Tidd